# Integrative multi-omics analysis identifies methylation-associated lncRNAs FAM83A-AS2 and AC012213.1 as candidate prognostic markers in lung adenocarcinoma

**DOI:** 10.1093/bioadv/vbag096

**Published:** 2026-07-14

**Authors:** Syed Muktadir Al Sium, Mahafujul Islam Quadery Tonmoy, Dhirendra Nath Barman, Jean-Christophe Nebel, Farzana Rahman, Sanjana Fatema Chowdhury

**Affiliations:** Bangladesh Council of Scientific and Industrial Research, Dhaka, 1205, Bangladesh; Bangladesh Council of Scientific and Industrial Research, Dhaka, 1205, Bangladesh; Department of Biotechnology & Genetic Engineering, Noakhali Science and Technology University, Noakhali, Bangladesh; Department of Biotechnology & Genetic Engineering, Noakhali Science and Technology University, Noakhali, Bangladesh; School of Computer Science and Mathematics, Faculty of Engineering, Computing and the Environment, Kingston University, London, KT1 2EE, United Kingdom; School of Computer Science and Mathematics, Faculty of Engineering, Computing and the Environment, Kingston University, London, KT1 2EE, United Kingdom; Bangladesh Council of Scientific and Industrial Research, Dhaka, 1205, Bangladesh

## Abstract

**Motivation:**

The Motivation: Long non-coding RNAs (lncRNAs) are important regulators of gene expression and are increasingly implicated in cancer. However, the extent to which aberrant DNA methylation is associated with lncRNA dysregulation in lung adenocarcinoma (LUAD) remains underexplored.

**Results:**

By integrating RNA-seq and Illumina 450K methylation data from 473 LUAD and 32 normal lung samples in TCGA, we identified 2,668 differentially expressed lncRNAs and 20,843 differentially methylated CpG sites. Mapping CpGs to lncRNA promoters (−1.5 kb from the annotated TSS) and Spearman correlation analysis highlighted seven lncRNAs with inverse promoter methylation–expression patterns; FAM83A-AS2 and AC012213.1 showed the most consistent associations and were linked to poorer overall survival in Kaplan–Meier and Cox models (adjusted for age, sex, and tumor stage). A two-lncRNA prognostic score derived using LASSO Cox regression showed moderate time-dependent discrimination at 590 days (AUC = 0.72) within the TCGA cohort. An exploratory lncRNA–miRNA–mRNA network suggested miRNA-mediated regulation of LUAD genes, with enrichment analyses linking targets to chromatin modification and epigenetic regulation. These findings nominate FAM83A-AS2 and AC012213.1 as candidate epigenetically associated prognostic lncRNAs in LUAD, warranting validation in independent cohorts and functional studies.

## Graphical abstract

**Graphical Abstract:**
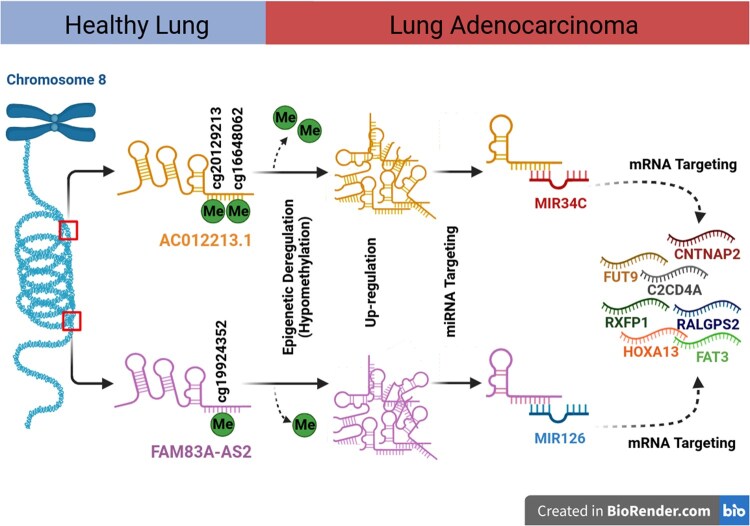

## 1 Introduction

Long non-coding RNAs (lncRNAs) are key regulators of gene expression and have emerged as potential cancer biomarkers (Slack and Chinnaiyan 2019). Epigenetic alterations, particularly DNA methylation, play a critical role in cancer by modifying gene expression without altering the DNA sequence (Sharma et al. 2010). In normal cells, DNA methylation is tightly controlled, but this process is disrupted in cancer, where promoter hypermethylation can silence tumour-suppressor genes, while hypomethylation can activate oncogenes (Baylin and Jones 2016).

The relationship between DNA methylation and lncRNA expression is a critical area of cancer research (Ranjbar et al. 2023). Lung adenocarcinoma (LUAD), a major subtype of lung cancer with high mortality, has a complex pathophysiology where epigenetically associated lncRNAs remain insufficiently characterized (Zappa and Mousa 2016). Here, we integrate transcriptomic and methylomic profiles to identify epigenetically associated lncRNAs in LUAD and evaluate their prognostic relevance as candidates for risk stratification.

## 2 Methods

### 2.1 Data sources and cohort selection

Multi-omics data were obtained from The Cancer Genome Atlas (TCGA) lung adenocarcinoma (LUAD) project via the UCSC Xena Browser and the Genomic Data Commons (GDC) (Tomczak et al. 2015). RNA-seq data were downloaded as raw gene-level read counts and DNA methylation data were downloaded as paired Illumina HumanMethylation450 (450K) IDAT files. From 585 LUAD specimens, 505 samples had matched expression and methylation profiles; downstream differential analyses were performed on 473 tumors and 32 normal lung samples. A complete list of the TCGA sample barcodes included in the study, together with sample type and data availability, is provided in Supplementary Table S1, available as supplementary data at *Bioinformatics Advances* online. Overall survival time, event status, and clinicopathologic variables (including age, sex, and stage) were obtained from TCGA clinical data.

### 2.2 RNA-seq processing and differential expression

Differential expression testing was conducted using DESeq2 (Love et al. 2014) with a tumor-versus-normal design on the raw count matrix. Genes with <10 total counts across all samples and genes on sex chromosomes (filtered manually from datasheet) were filtered prior to modeling. DESeq2 size-factor normalization and negative-binomial generalized linear modeling were used for hypothesis testing; P-values were adjusted using the Benjamini–Hochberg false discovery rate (FDR). Genes/lncRNAs with FDR < 0.05 and |log2FC| > 1.5 were considered differentially expressed. Variance-stabilizing transformation (VST) was used for downstream correlation analyses and visualization.

### 2.3 DNA methylation processing and differential methylation

IDAT files were processed with the ChAMP pipeline (Tian et al. 2017). Standard probe-level QC and filtering followed the pipeline defaults used in our original analysis: probes with detection P > 0.01 were removed; probes with bead count < 3 in at least 5% of the samples were excluded; non-CpG probes, SNP-associated probes, multi-hit/cross-reactive probes, and probes on sex chromosomes were filtered out. After QC, 365,186 probes across 505 samples were retained for downstream analyses. Beta-mixture quantile (BMIQ) normalization was applied to reduce type I/II probe bias. Differentially methylated CpGs (DMCs) between tumor and normal samples were identified using ChAMP’s champ. DMP function (type–categorical). Benjamini–Hochberg method was applied for multiple testing correction of raw p-values to control the false discovery rate (FDR). CpG sites with FDR < 0.05 and absolute delta beta (|Δβ|) > 0.2 were considered significant DMCs. No batch correction was applied; potential impacts of cohort imbalance and batch effects are discussed as limitations.

### 2.4 Integration of methylation and lncRNA expression

DMCs were mapped to promoters of differentially expressed lncRNAs using Illumina 450K hg38 manifest annotations (for CpG genomic coordinates) and transcript coordinates for lncRNAs. Promoters were defined as the 1500 bp region upstream of the transcription start site (TSS). Spearman rank correlations were computed for each CpG–lncRNA pair between promoter CpG beta values and VST-transformed lncRNA expression across LUAD tumors, after confirming non-normality using the Shapiro–Wilk test. Candidate epigenetically deregulated lncRNAs were required to show promoter hypomethylation in tumors and an inverse methylation–expression association (*ρ* < −0.4, nominal *P* < 0.05).

### 2.5 Survival modeling and risk-score evaluation

After merging molecular and clinical data and excluding missing/invalid survival entries, *N* = 473 patients were included in survival analyses. Patients were stratified into high- and low-expression/methylation groups for each candidate lncRNA using the cohort median. Kaplan–Meier survival curves and Cox proportional hazards models (Efron 1988) were applied using the “survival” (Therneau and Lumley 2015) and “survminer” (Kassambara et al. 2017) R packages to evaluate overall survival associations. Multivariable Cox models were adjusted for age, sex, and pathologic stage, with statistical significance defined by hazard ratio (HR) 95% confidence intervals and P < 0.05. To derive a parsimonious prognostic signature, LASSO-penalized Cox regression was performed using glmnet (Friedman et al. 2021) with α  =  1 and maxit = 1000, with cross-validation used to select optimal λ (λmin and λ1se). Individual risk scores were calculated as the linear predictor of the fitted model. Prognostic performance was assessed using time-dependent ROC analysis via “survivalROC” (Heagerty et al. 2013) at 590 days (predict.time = 590), corresponding to the cohort’s median survival time, using the nearest-neighbor estimator (method = “NNE”).

### 2.6 Regulatory network construction and functional analysis

Putative lncRNA–miRNA interactions were obtained from miRcode (Jeggari et al. 2012). Only differentially expressed miRNAs (DEmiRNAs) were retained for further analysis. miRNA–mRNA interactions were retrieved using miRWalk (Sticht et al. 2018). Downstream targets were restricted to differentially expressed protein-coding genes shared by the retained DEmiRNAs. For correlation-based prioritization, a pre-filtered expression matrix containing the prioritized DElncRNAs and these DEmRNAs was analyzed. Pairwise Spearman correlations were computed across this matrix using Hmisc (Harrell Jr & Harrell Jr, 2019) (*ρ* > 0.2, *P* < 0.05), but only lncRNA–mRNA correlations were used for downstream biological interpretation and core network definition. As an exploratory prioritization step following prior filtering, correlation P-values were not adjusted for multiple testing and should therefore be interpreted cautiously. The lncRNA–miRNA–mRNA network was assembled and visualized in Cytoscape (Kohl et al. 2011) using default settings. Functional enrichment of network genes was performed using DAVID (Dennis et al. 2003), and a chord diagram linking genes to GO molecular functions was produced using GOplot (Walter et al. 2015). Pre-ranked gene set enrichment analysis (GSEA) was conducted using the GSEA software (Subramanian et al. 2005) with log2FC as the ranking metric, gene sets from the MSigDB C2 curated collection, 1000 permutations (seed set to “Timestamp”), and the “weighted” enrichment statistic with “meandiv” normalization.

An overview of the workflow is shown in Supplementary Figure S1, available as supplementary data at *Bioinformatics Advances* online.

## 3 Results

**Figure 1:**
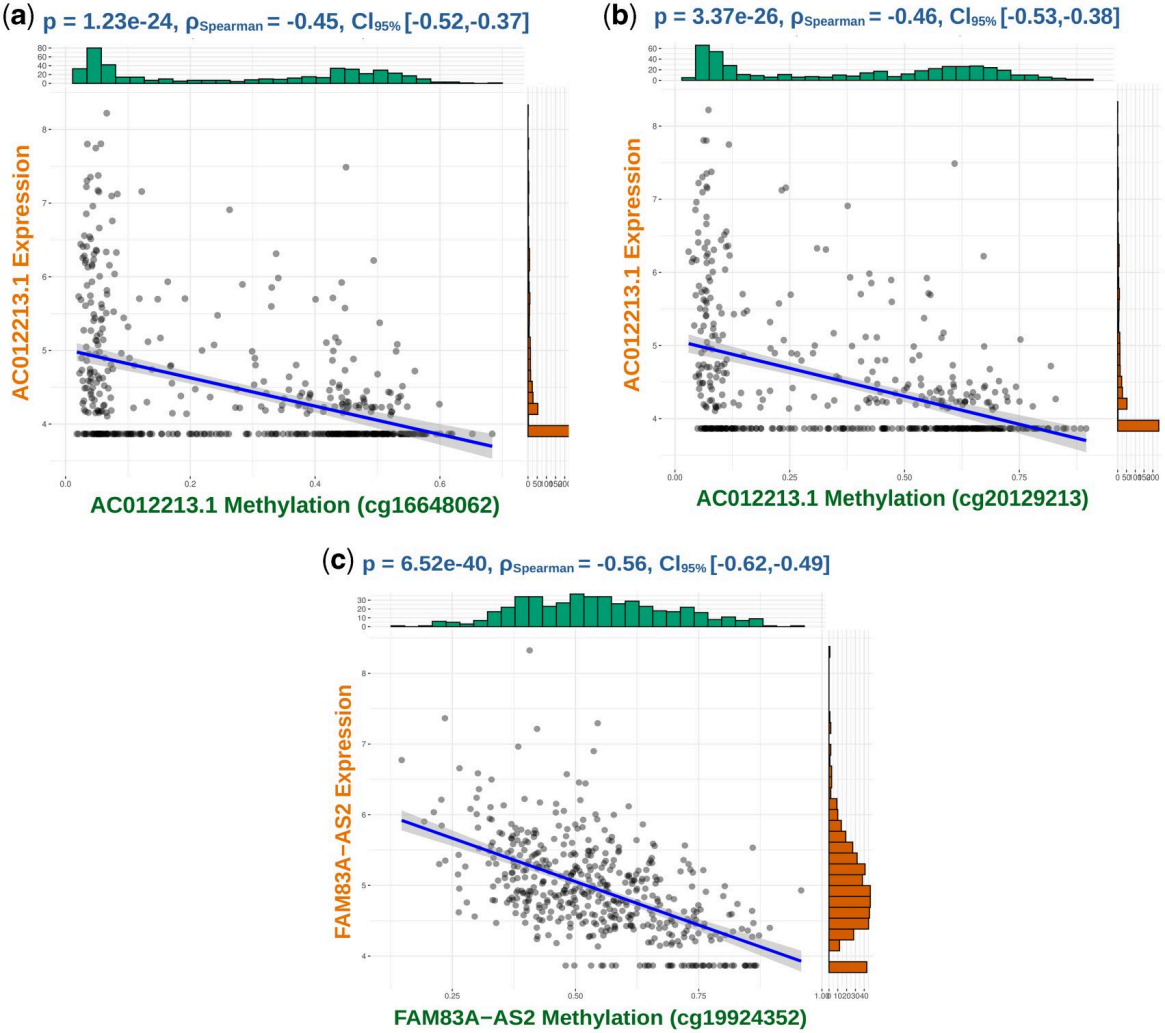
Correlation between promoter methylation and expression for key DElncRNAs. A significant negative correlation is observed between the expression of (a) AC012213.1 and methylation of cg16648062, (b) AC012213.1 and cg20129213, and (c) FAM83A-AS2 and cg19924352.

Differential expression and methylation analyses identified 8,327 DEGs, including 2,668 DElncRNAs (Supplementary Figures S2, S3; Supplementary Table S2, available as supplementary data at *Bioinformatics Advances* online) and 20,843 differentially methylated CpG sites (Supplementary Table S3, available as supplementary data at *Bioinformatics Advances* online). Consistent with established cancer methylation patterns, hypermethylation was enriched in CpG islands, whereas hypomethylation was more frequent in open-sea regions (Supplementary Figure S4, available as supplementary data at *Bioinformatics Advances* online). Integrating promoter-proximal CpGs with DElncRNAs highlighted seven lncRNAs with inverse promoter methylation–expression patterns (*ρ* < −0.4; nominal *p* < 0.05) across tumors (Supplementary Figure S6; Supplementary Table S4, available as supplementary data at *Bioinformatics Advances* online). Among these, FAM83A-AS2 and AC012213.1 showed the most prominent promoter hypomethylation and inverse CpG–expression correlations at cg19924352 (FAM83A-AS2) and cg16648062& cg20129213 (AC012213.1) (Fig. 1). Correlation plots for the remaining candidates are provided in Supplementary Figure S5, available as supplementary data at *Bioinformatics Advances* online.

**Figure 2:**
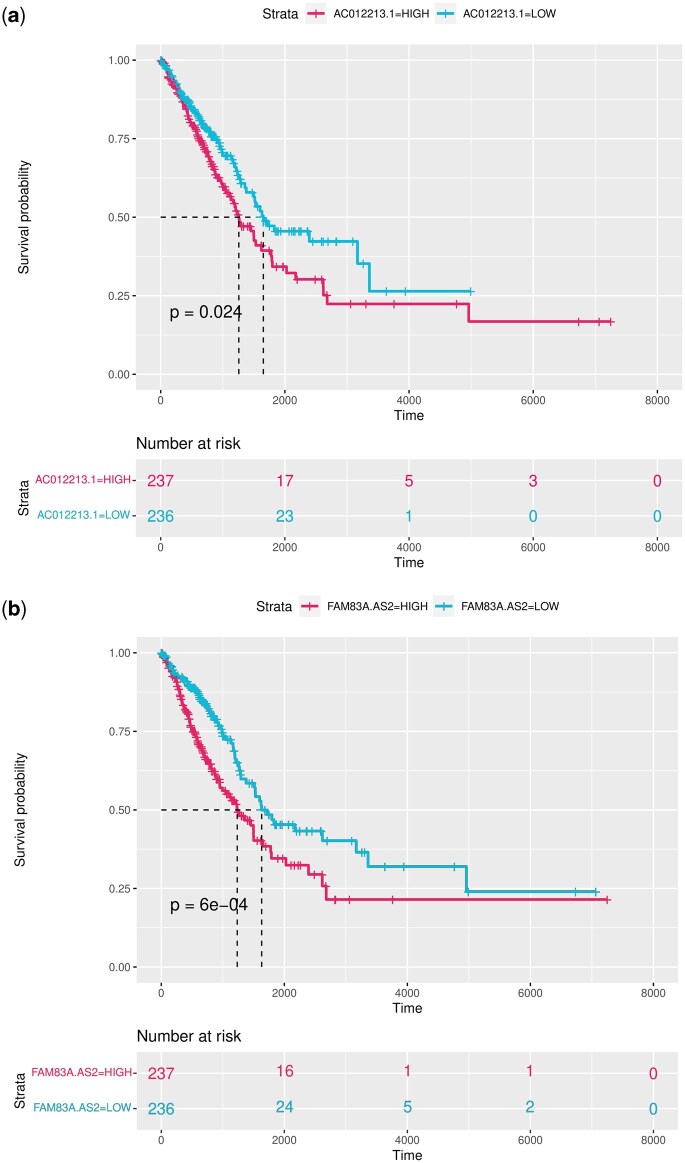
Kaplan-Meier survival curves for LUAD patients. High expression of (a) AC012213.1 (*p* = 0.024) and (b) FAM83A-AS2 (*p* = 6e-04) is associated with significantly poorer overall survival.

To assess clinical relevance, we performed univariate survival analyses for the seven candidate DElncRNAs and their promoter-associated CpGs. Only FAM83A-AS2 and AC012213.1 (and their associated CpGs) showed associations with poorer overall survival (*p* < 0.05; Hazard Ratio > 1) in this screening step (Supplementary Figure S7, available as supplementary data at *Bioinformatics Advances* online) and were prioritized for downstream modeling. Kaplan–Meier analysis showed reduced overall survival among patients with higher expression of either lncRNA (Fig. 2).

To estimate independence from key clinical covariates, we fitted a multivariate Cox model adjusting for age, sex, and tumor stage. High FAM83A-AS2 expression remained associated with poorer survival (HR 1.55, 95% CI 1.13–2.13; Table 1). AC012213.1 showed a more modest association (HR 1.30) with a borderline 95% CI overlapping 1 (0.95–1.78), and is therefore interpreted cautiously (Table 1).

**Table 1:** Multivariate Cox-regression analysis for overall survival in LUAD patients.

Variable	Hazard Ratio	95% Confidence Interval	*P*-value
Age (>65 vs <65)	0.77	0.56–1.05	0.10
Gender (male vs female)	0.89	0.65–1.21	0.47
Tumor Stage (III+IV vs I+II)	2.21	1.59–3.08	2.25e-06
**FAM83A-AS2 (high vs low)**	**1.55**	**1.13–2.13**	**0.0063**
**AC012213.1 (high vs low)**	**1.30**	**0.95–1.78**	**0.021**

**Figure 3:**
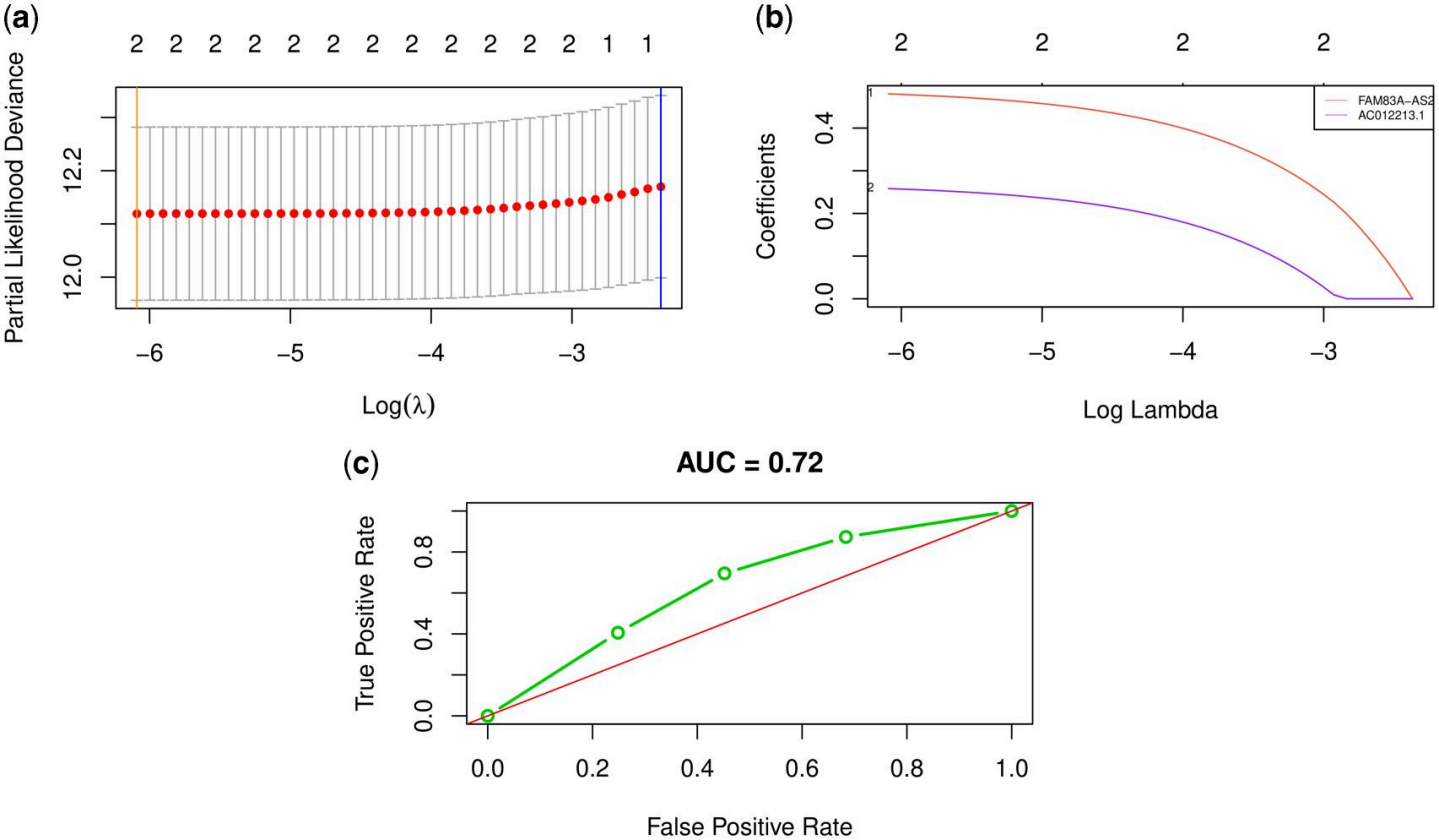
Prognostic modeling of the two-lncRNA signature. (a) Cross-validated partial likelihood deviance used to select the LASSO penalty. (b) Regularization path showing non-zero coefficients for FAM83A-AS2 and AC012213.1. (c) Time-dependent ROC at 590 days for the TCGA-derived risk score (AUC = 0.72).

We next derived a parsimonious prognostic score using LASSO Cox regression. Both lncRNAs retained non-zero coefficients at the cross-validated optimal penalty (0.48 for FAM83A-AS2 and 0.26 for AC012213.1; Fig. 3a–b). Using the resulting linear predictor as a risk score, time-dependent ROC analysis at 590 days yielded an AUC of 0.72 (Fig. 3c), indicating moderate discrimination for survival outcome within the TCGA cohort. Because model development and evaluation were performed in the same dataset, these estimates should be considered exploratory and require independent validation or nested cross-validation in future work.

**Figure 4:**
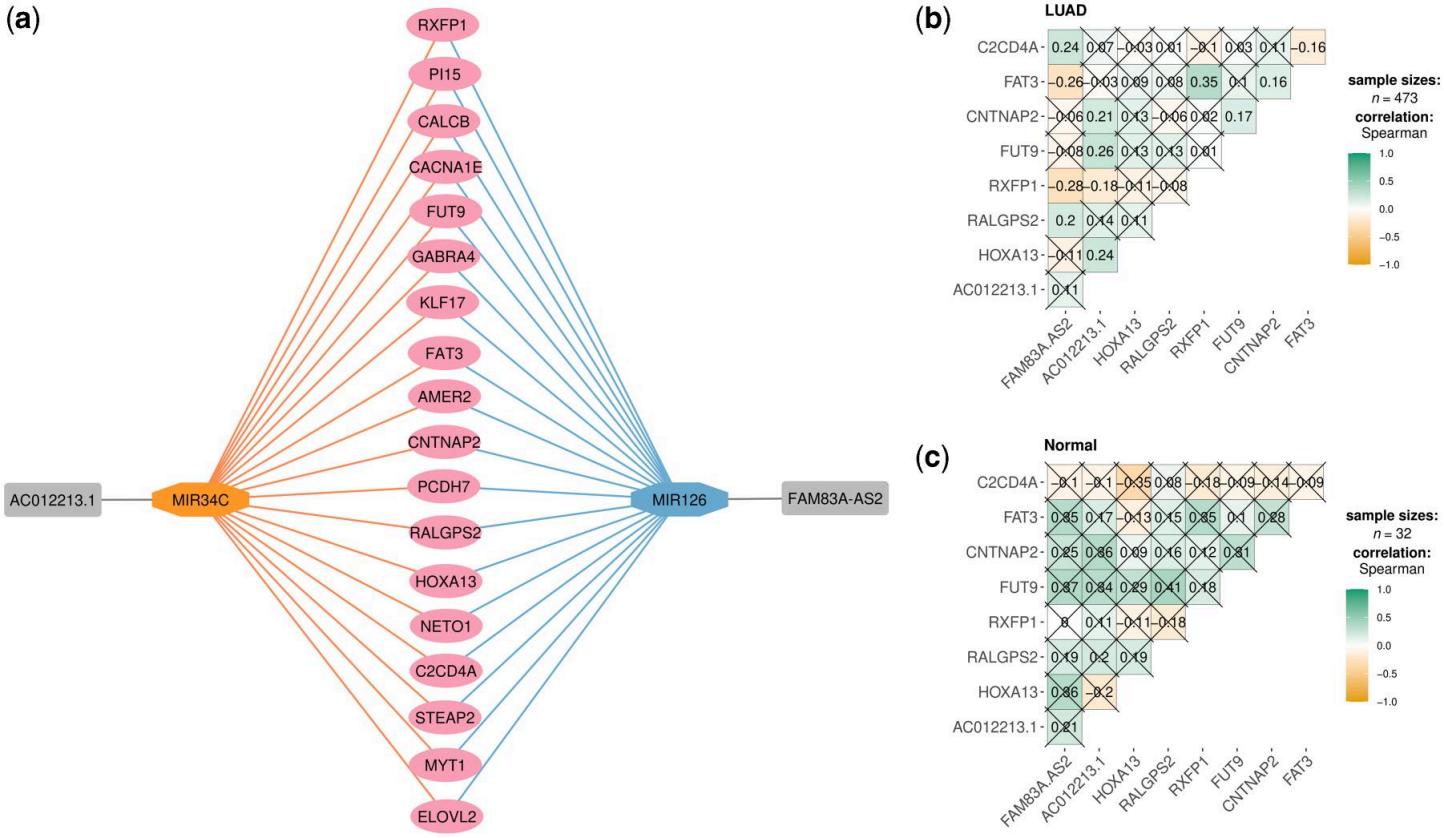
The lncRNA-miRNA-mRNA regulatory network with correlation matrix. (a) The epigenetically deregulated lncRNAs FAM83A-AS2 and AC012213.1 regulate key LUAD-associated mRNAs through interactions with MIR126 and MIR34C. (b) Spearman’s correlation matrix depicting the correlations between the two key DElncRNAs and the seven core DEGs in LUAD samples. (c) The same correlation matrix for normal samples, where correlations are not significant.

To explore potential downstream context, we constructed an lncRNA–miRNA–mRNA network. miRcode predicted 16 miRNAs targeting FAM83A-AS2 and/or AC012213.1; intersecting these with our differential expression results retained two DEmiRNAs (MIR126 and MIR34C). miRWalk predicted targets of these miRNAs, yielding thousands of candidate mRNAs (full edge list is provided in the Supplementary Table S5, available as supplementary data at *Bioinformatics Advances* online); restricting to LUAD DEGs retained 18 DEmRNAs shared by both miRNAs (Fig. 4a). All pairwise correlations within this pre-filtered 20-variable matrix (2 lncRNAs and 18 DEmRNAs) were calculated; however, only lncRNA–mRNA pairs were carried forward for biological interpretation and network refinement. Correlation analysis between two lncRNAs and these 18 DEmRNAs highlighted 7 mRNAs (C2CD4A, RALGPS2, RXFP1, FAT3, CNTNAP2, FUT9, and HOXA13) with significant expression associations in tumors (Fig. 4b; lack of corresponding associations in normals is shown in Fig. 4c).

Functional enrichment analyses provided biological context for the core lncRNA–miRNA–mRNA network identified in LUAD. Gene Ontology analysis of the seven network mRNAs highlighted transcriptional regulation, chromatin organization, and signal transduction–related functions, processes that are frequently dysregulated during LUAD progression (Supplementary Figure S8; Supplementary Table S6, available as supplementary data at *Bioinformatics Advances* online). Gene set enrichment analysis further showed that higher expression of FAM83A-AS2 and AC012213.1 was associated with chromatin modification and DNA methylation–related gene sets (Supplementary Figure S9, available as supplementary data at *Bioinformatics Advances* online), consistent with the established role of epigenetic deregulation in lung adenocarcinoma. Notably, several network genes have prior links to cancer biology: FUT9 has been implicated in aberrant fucosylation affecting tumor–immune interactions and metastatic behavior (Gao et al. 2022), HOXA13 is a developmental transcription factor associated with oncogenic reprogramming and poor prognosis in multiple cancers (Deng et al. 2018), and RalGPS2 is essential for survival and cell cycle progression of lung cancer cells (Santos et al. 2016). The LUAD-specific correlations between these genes and the two lncRNAs suggest a context-dependent regulatory program, supporting their potential relevance as epigenetically associated biomarkers rather than generic transcriptional changes. Given the reliance on *in silico* predictions, the network is presented as a mechanistic hypothesis. Notably, FAM83A-AS2 is located in close genomic proximity to FAM83A-AS1, a lncRNA previously reported to promote LUAD cell proliferation and tumor progression (Chen et al. 2022). Although FAM83A-AS1 was not directly analyzed in the present study, this positional relationship provides additional biological context supporting the potential oncogenic relevance of the FAM83A-AS locus in LUAD.

## 4 Conclusion

This study integrates TCGA transcriptomic and 450K methylation profiles to nominate two lncRNAs, FAM83A-AS2 and AC012213.1, whose expression is inversely associated with promoter methylation and with overall survival in LUAD. Within the TCGA cohort, a two-lncRNA score showed moderate time-dependent discrimination, and exploratory network/enrichment analyses point to epigenetic and chromatin-related pathways. Limitations include the tumor–normal sample imbalance, lack of external validation, and the absence of explicit batch-effect correction; therefore, the findings should be interpreted as associations that motivate follow-up studies. Future work should validate these candidates in independent cohorts and test functional links between promoter methylation and lncRNA expression experimentally.

## Supplementary Material

vbag096_Supplementary_Data

## Data Availability

All data analyzed are publicly available from TCGA (RNA-seq counts via UCSC Xena; 450K IDATs and clinical/survival data via the GDC portal). Processed result tables are provided as Supplementary Tables. Consolidated analysis scripts are publicly available via a GitHub repository (https://github.com/sium007/BCSIR-LUAD-Study).
