# Supplementary material for: Integrative multi-omics analysis identifies methylation-associated lncRNAs FAM83A-AS2 and AC012213.1 as candidate prognostic markers in lung adenocarcinoma
Source: Bioinform Adv. 2026 Jul 14;6(1):vbag096. doi: 10.1093/bioadv/vbag096 (PMC13384057; doi:10.1093/bioadv/vbag096)
Supplement: vbag096_Supplementary_Data [file vbag096_supplementary_data.zip › Attached file_ supplementary-files-updated-v2.docx]

# Supplementary Figures

**Supplementary Figure S1:** Methodology Flowchart


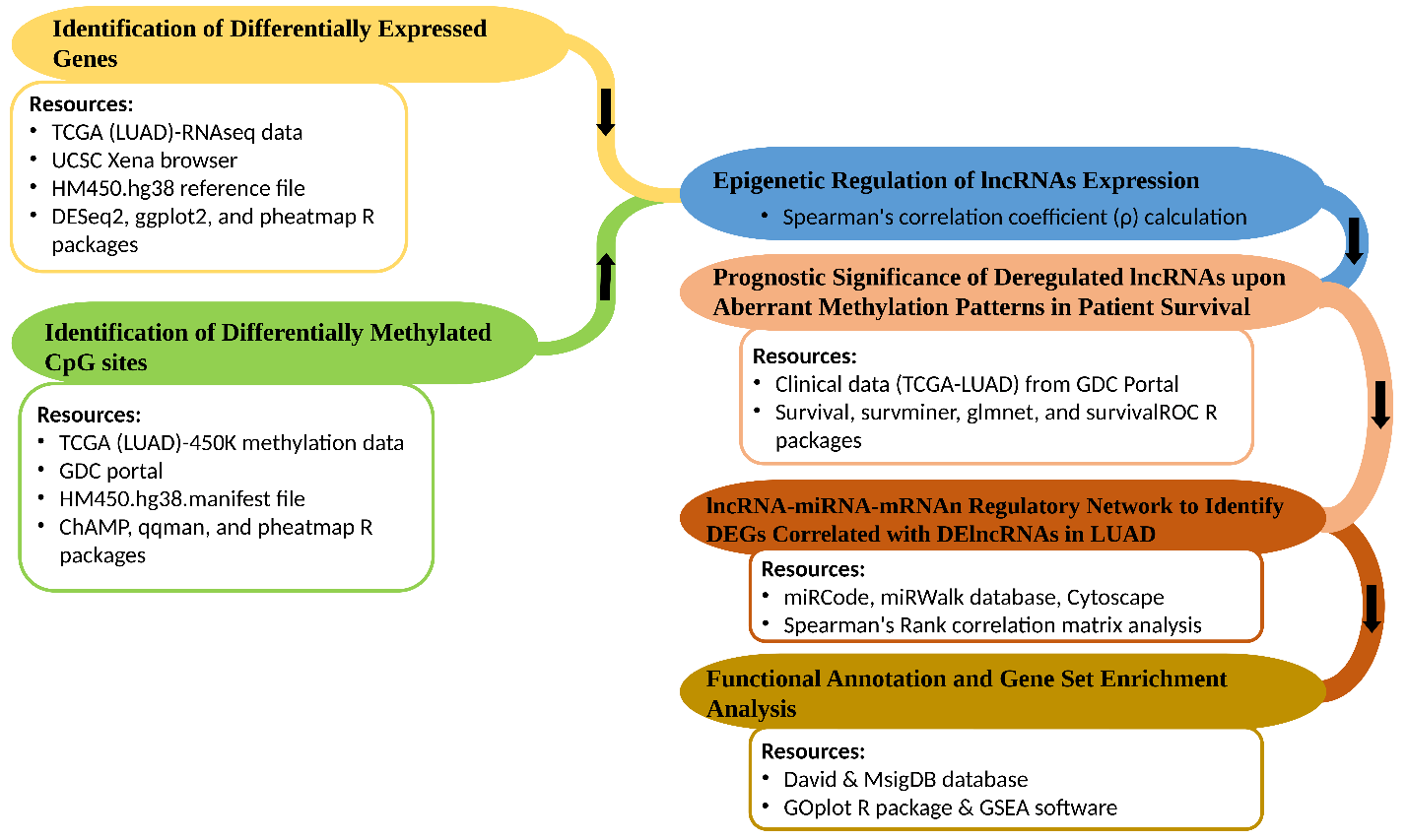


A diagram illustrating the complete research methodology and resources used in this study.

**Supplementary Figure S2:** Genome-wide Expression Patterns of Genes in LUAD
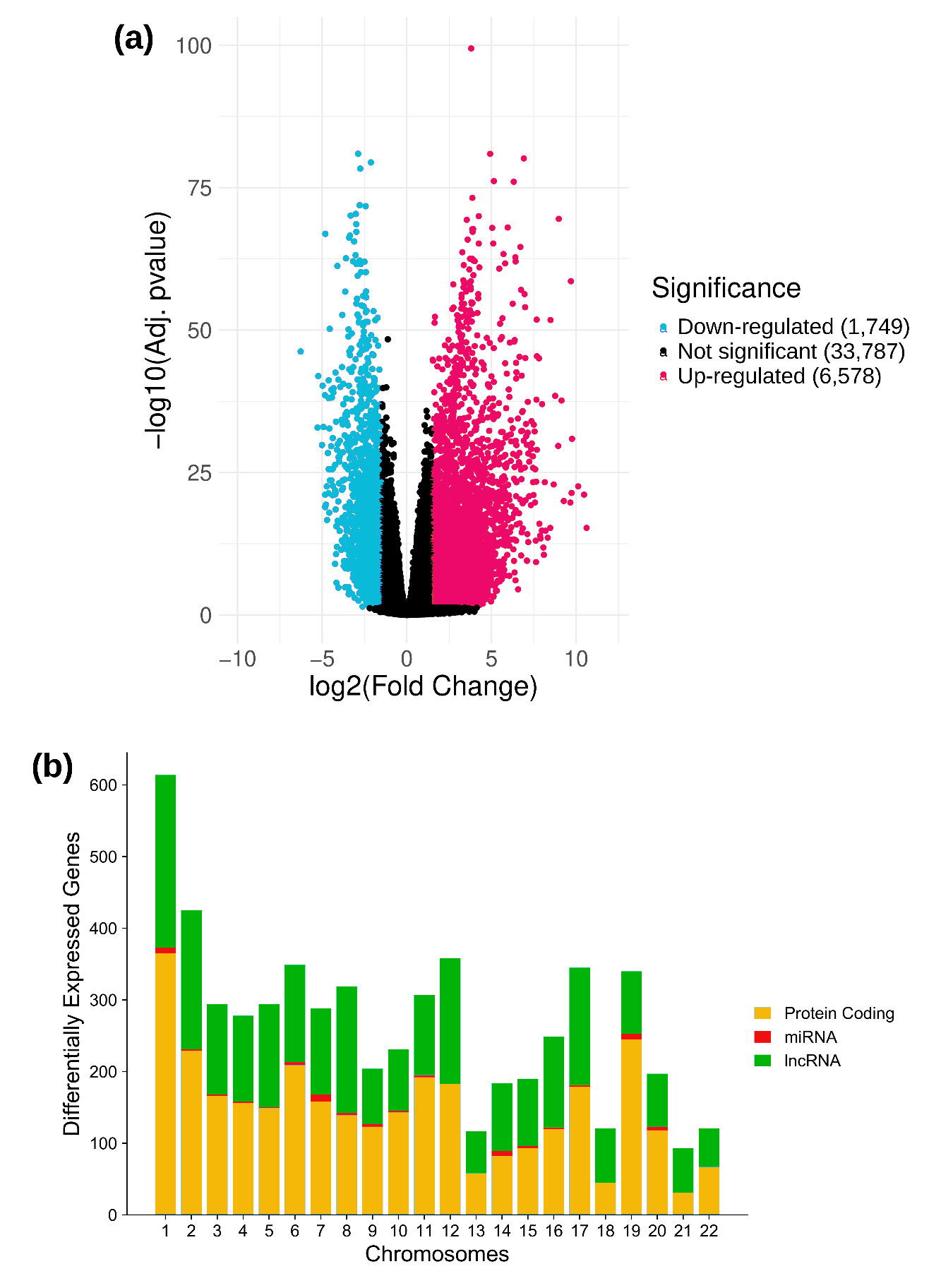


(a) A volcano plot showing differentially expressed genes. (b) A bar plot showing the distribution and types of differentially expressed genes across autosomal chromosomes.

**Supplementary Figure S3:** Genome-wide Expression Patterns of lncRNAs in LUAD


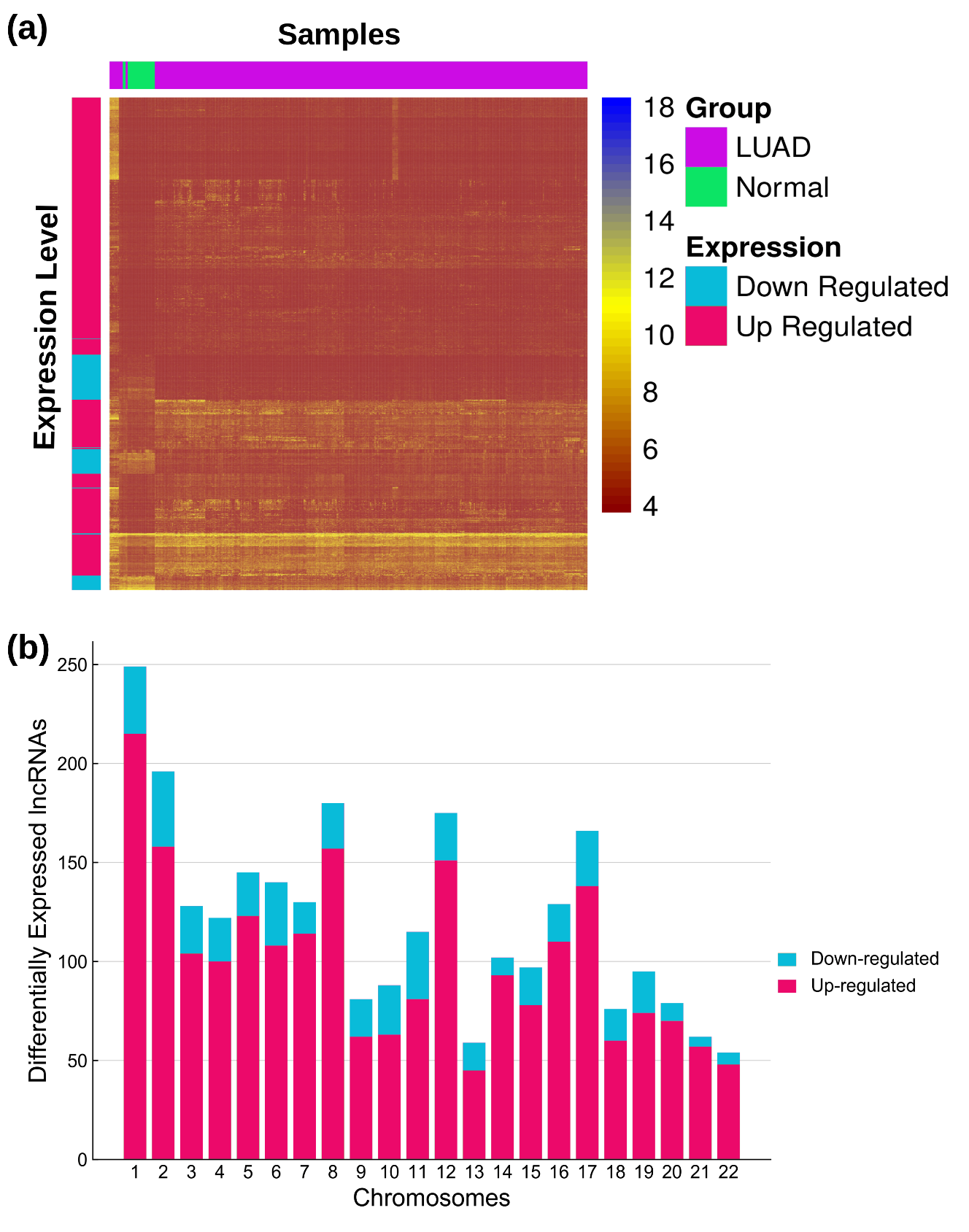


1. A heatmap showing the differential expression pattern of lncRNAs between LUAD and normal samples. (b) A bar plot showing the distribution of up- and down-regulated lncRNAs across autosomal chromosomes.

**Supplementary Figure S4:** Genome-wide Methylation Patterns of CpG Sites in LUAD


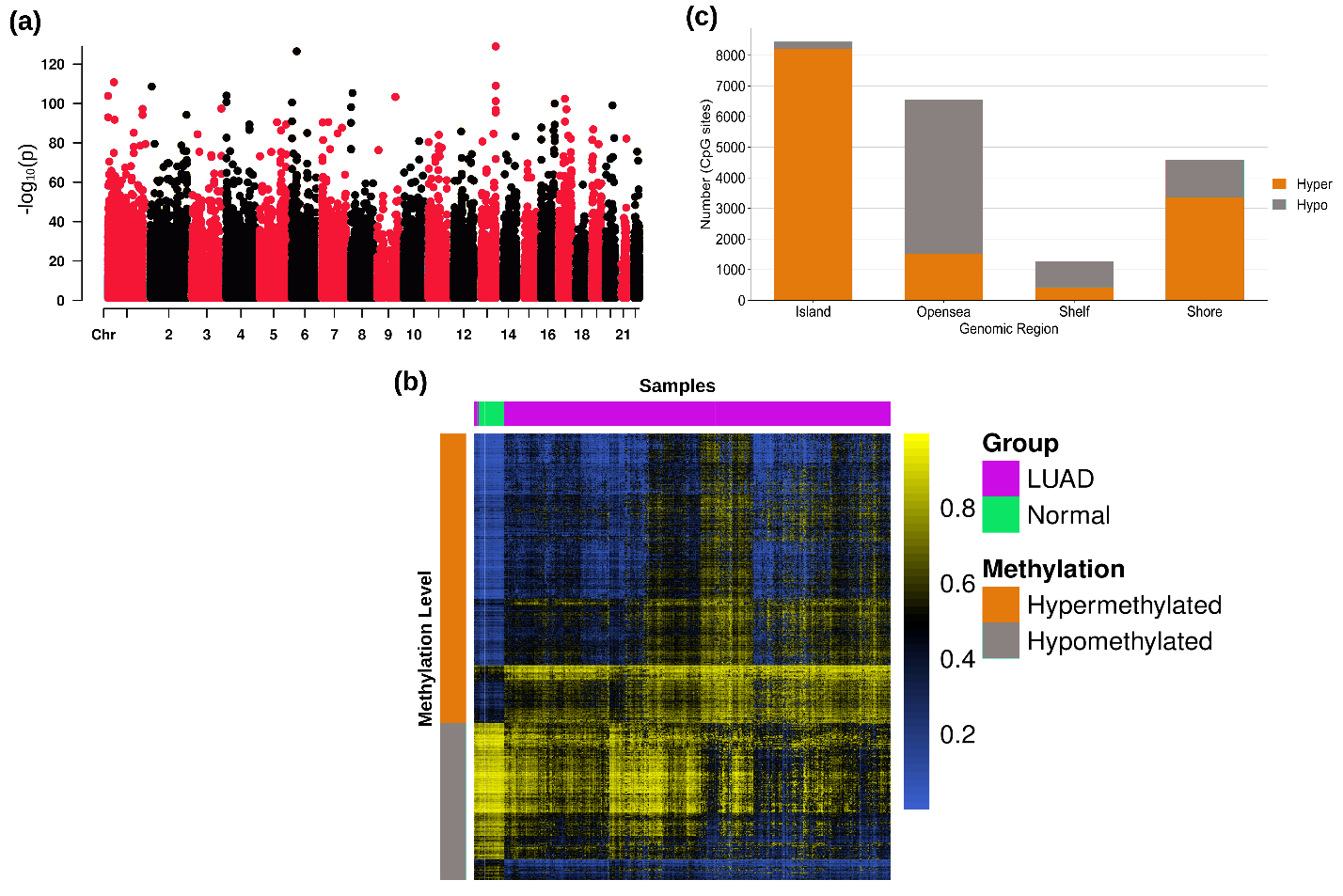


1. A Manhattan plot displaying the chromosomal distribution of CpG sites. (b) A heatmap of absolute methylation β-values (0–1) for significant DMCs across samples. Hyper / hypomethylation row labels are based on Δβ thresholds from the differential methylation analysis. (c) A bar plot showing the distribution of hyper- and hypo-methylated sites across different genomic regions (Island, Opensea, Shelf, Shore).

**Supplementary Figure S5:** Correlation Plots for Additional Candidate DElncRNAs


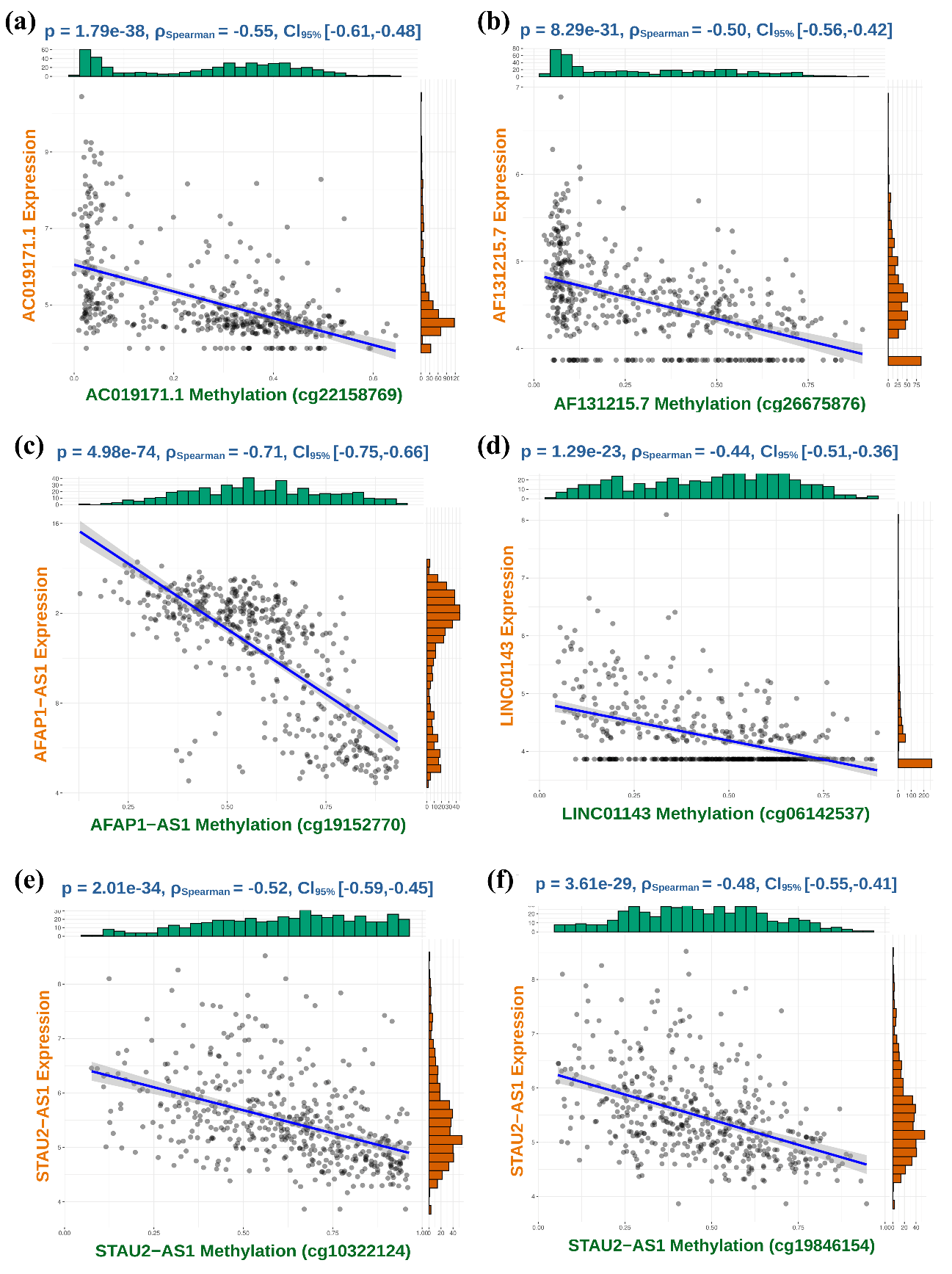


Correlation plots showing the relationship between promoter methylation and expression for (a) AC019171.1, (b) AF131215.7, (c) AFAP1-AS1, (d) LINC01143, and (e & f) STAU2-AS1.

**Supplementary Figure S6:** Expression and Methylation Patterns of Seven Epigenetically Deregulated DElncRNAs


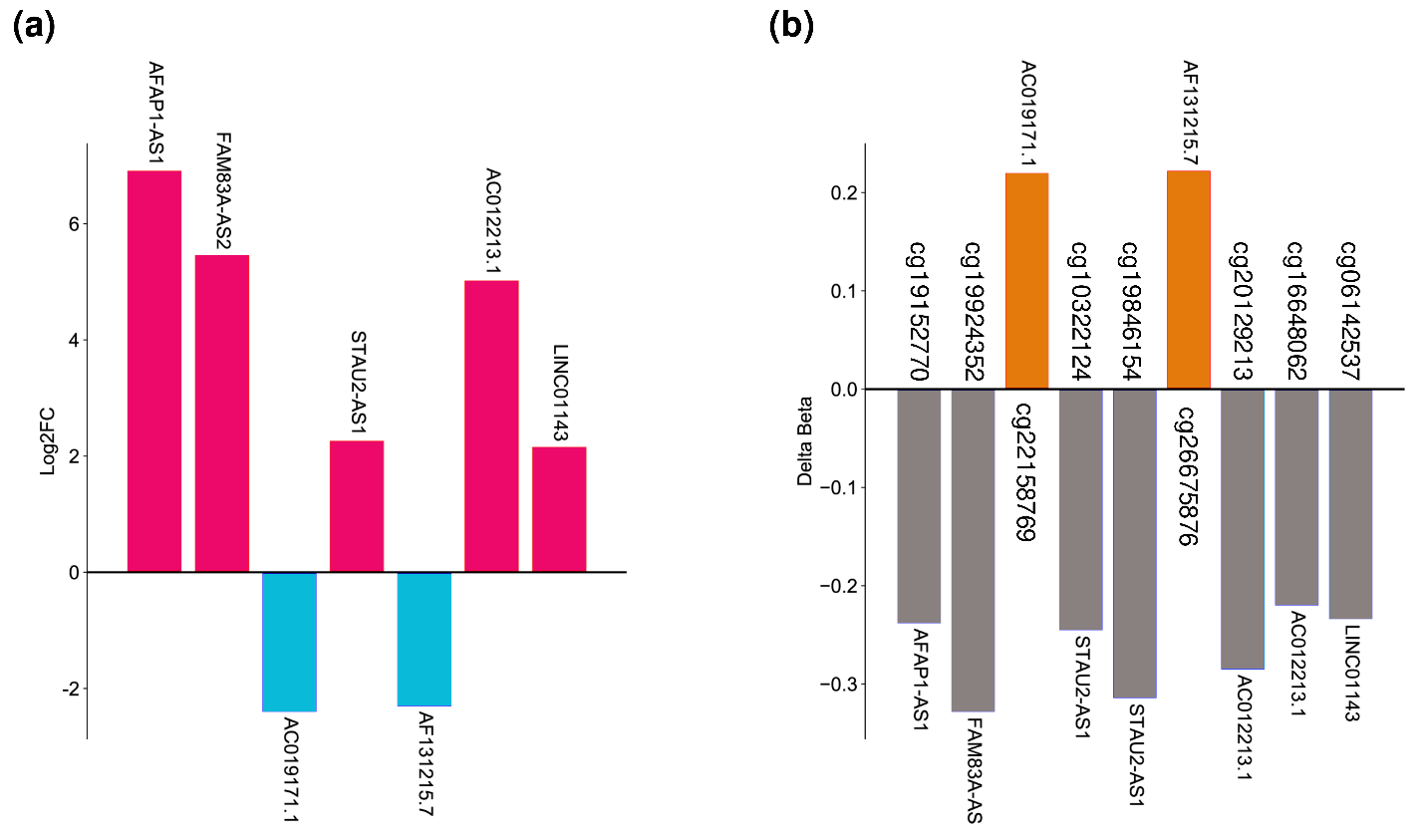


(a) Bar plot showing the Log2 Fold Change for the seven DElncRNAs. (b) Bar plot showing the Delta Beta values for the nine associated DMCs in the promoter regions of these lncRNAs.

**Supplementary Figure S7:** Forest Plot for Univariate Cox Regression Analysis


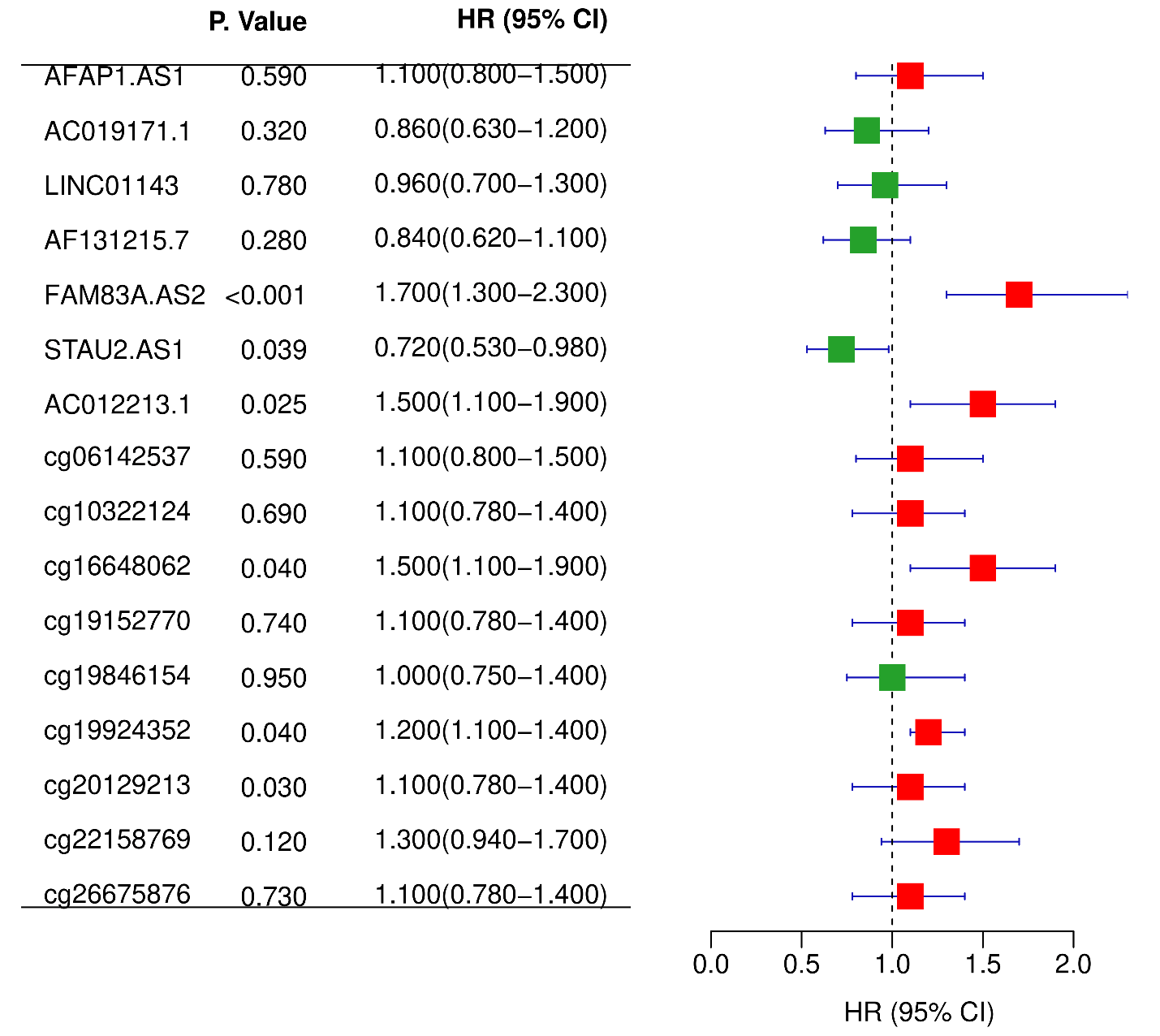


This plot shows the Hazard Ratios (HR) and 95% Confidence Intervals for the seven candidate DElncRNAs and nine DMCs. FAM83A-AS2, AC012213.1, and their associated DMCs show a statistically significant (p < 0.05) association with increased risk (HR > 1).

**Supplementary Figure S8:** Gene Ontology Molecular Function Chord Plot


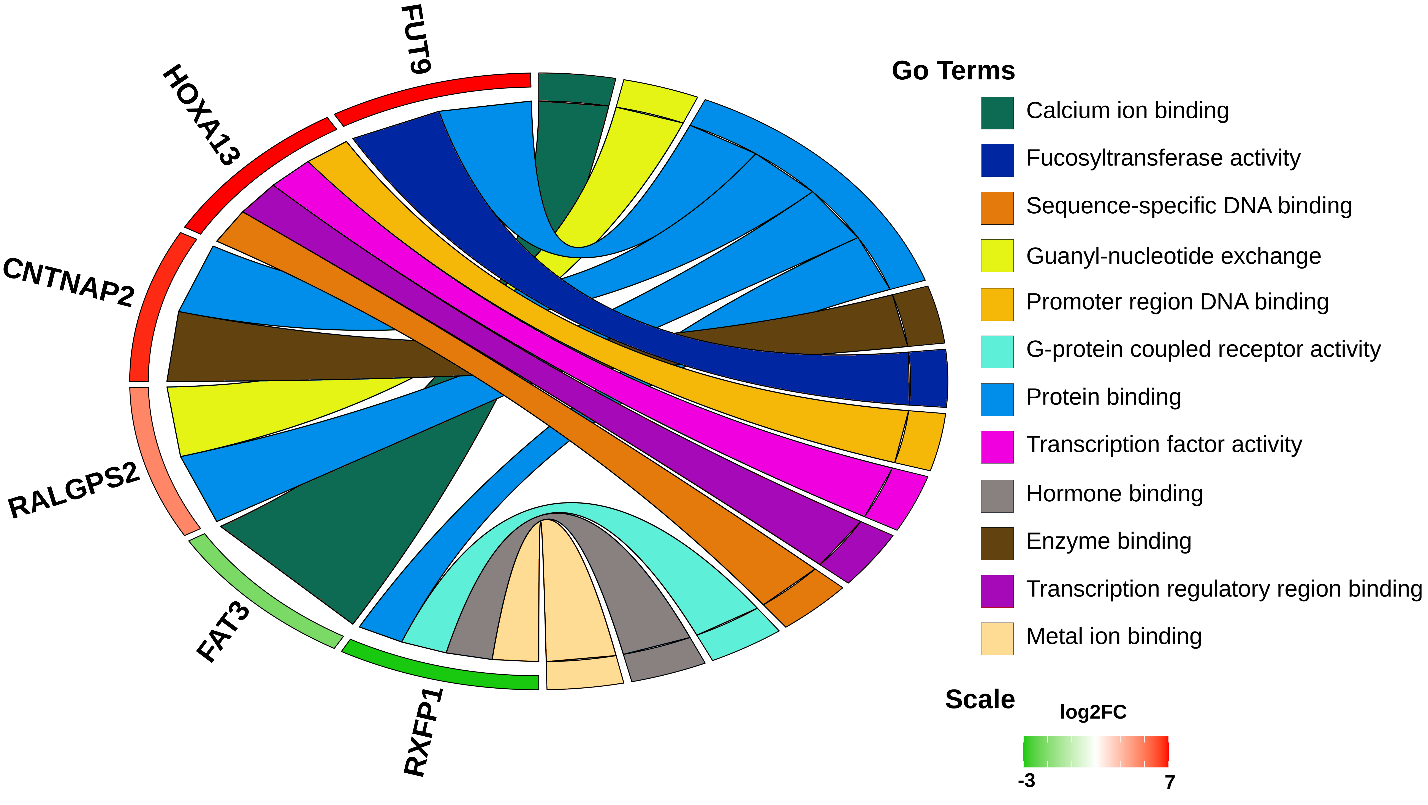


A chord diagram illustrating the molecular functions associated with the core DEGs, such as Fucosyltransferase activity, Transcription factor activity, and Protein binding.

Supplementary Figure S9: Gene Set Enrichment Analysis (GSEA) Enrichment Plots


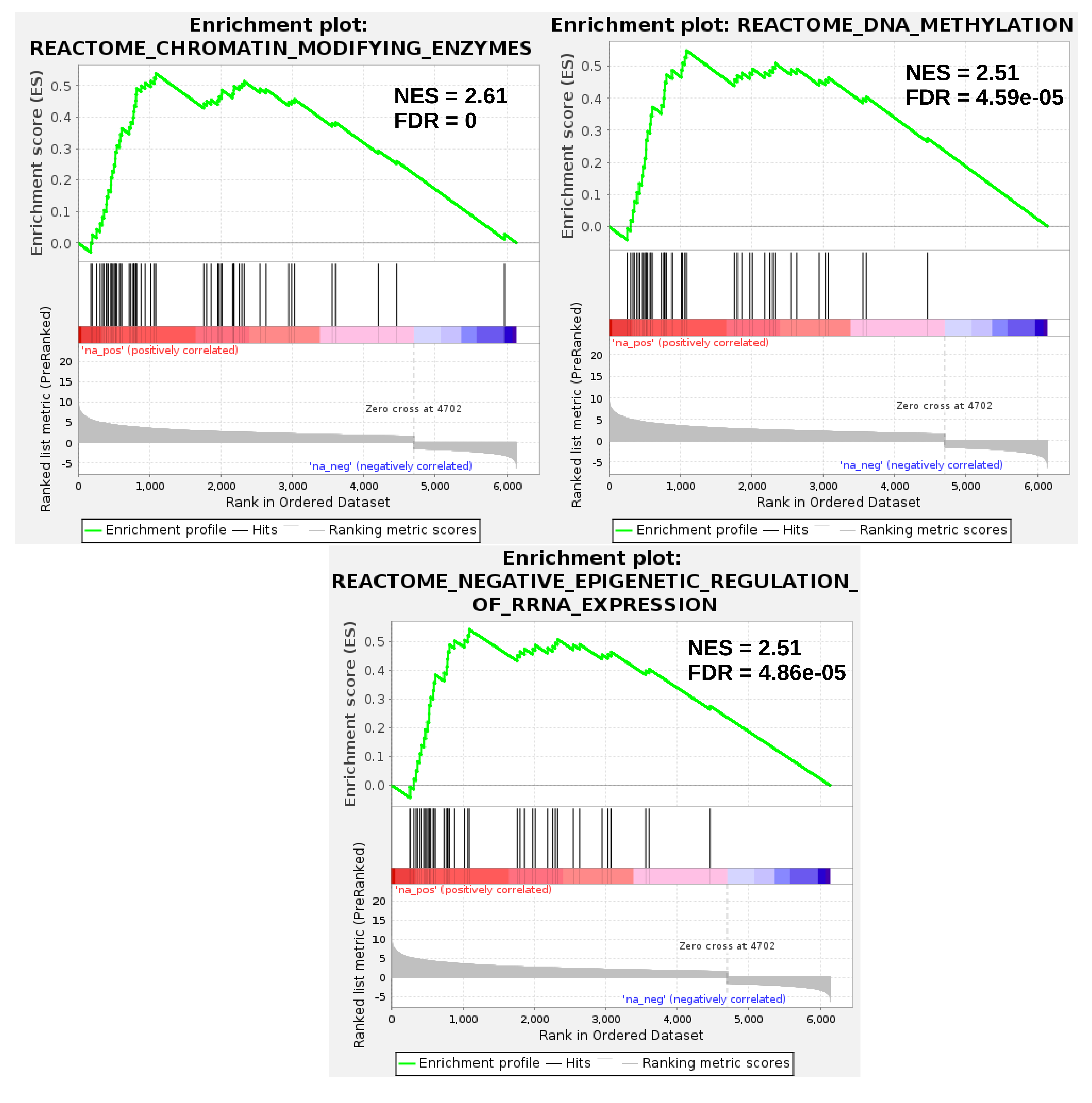


Enrichment plots from GSEA demonstrating the significant positive correlation of FAM83A-AS2 and AC012213.1 expression with (a) Chromatin Modifying Enzymes, (b) DNA Methylation, and (c) Negative Epigenetic Regulation of rRNA Expression. A positive normalized enrichment score (NES) indicates a positive correlation with the phenotype profile.

# Supplementary Tables

**Supplementary Table S1:** TCGA Sample Barcodes and Data Availability

A complete list of TCGA sample barcodes included in the study, together with sample type (LUAD/normal) and the availability of RNA-seq and DNA methylation data.

**Supplementary Table S2:** Differentially Expressed lncRNAs in LUAD

A complete list of the 2,668 differentially expressed lncRNAs identified between LUAD and normal samples, including their Log2 Fold Change and adjusted p-values.

**Supplementary Table S3:** Differentially Methylated CpG Sites in LUAD

A complete list of the 20,843 differentially methylated CpG sites (DMCs), including their chromosomal location, Delta Beta values, and adjusted p-values.

**Supplementary Table S4:** Negatively Correlated DMC-DElncRNA Pairs

A list of the 156 DMCs that showed a significant negative correlation with the expression of 84 DElncRNAs, including correlation coefficients and p-values.

**Supplementary Table S5:** Target Genes of Differentially Expressed miRNAs

A comprehensive list of protein-coding genes targeted by the differentially expressed miRNAs, MIR126 and MIR34C.

**Supplementary Table S6:** Gene Ontology Molecular Function Analysis Results

Detailed results from the DAVID analysis for the core set of seven DEGs, including GO terms, p-values, and associated genes.
